# Cancer Morbidity in Rheumatoid Arthritis: Role of Estrogen Metabolites

**DOI:** 10.1155/2013/748178

**Published:** 2013-09-17

**Authors:** Wahid Ali Khan, Mohd Wajid Ali Khan

**Affiliations:** ^1^Department of Clinical Biochemistry, College of Medicine, King Khalid University, Abha 641, Saudi Arabia; ^2^Institute of Infection and Immunity, Cardiff University School of Medicine, Henry Wellcome Building, Heath Park, Cardiff CF14 4XN, UK

## Abstract

Estrogen metabolites have been implicated in rheumatoid arthritis (RA) and cancer, although the mechanism remains unestablished. Some estrogen metabolites, which are used for the assessment of cancer risk, play an important role in RA. The pathways by which malignancies associated with RA remain elusive. Possible mechanism involves enzymatic or nonenzymatic oxidation of estrogen into catecholestrogen metabolites through semiquinone and quinone redox cycle to produce free radicals that can cause DNA modifications. Modifications of DNA alter its immunogenicity and trigger various immune responses leading to elevated levels of cancer and RA antibodies. However, the role of different estrogen metabolites as a mediator of immune response cannot be ruled out in various immune-related diseases.

## 1. Introduction

The associations of malignancy with autoimmune rheumatic diseases have been well established, but none of the studies reported any evidence regarding the role of estrogen metabolites in cancer morbidities in RA patients. It has been found that certain cancers are more common, some are less common, and some are the same as for non-RA population. It is interesting to know that measurement of some estrogen metabolites for the assessment of cancer risk could play important role in RA. RA is an autoimmune condition in which dysregulated lymphocytes react against self-antigens by producing autoantibodies, and the normal immune function is suppressed [1]. Dysregulations of the host's immune surveillance as well as immunosuppression are the risk factors for different types of cancer [2]. RA has been known to increase the risk of lymphoma [3] along with other types of cancer. These patients are at high risk for lymphoma, while lymphoma patients are not at high risk for RA. And the more severe the RA is, the higher the risk of lymphoma is. In addition, there are almost 8 different types of cancer linked to RA, which may be caused by RA medications or RA-related inflammation itself. These include lung, skin, myeloma, non-Hodgkin's lymphoma and Hodgkin's disease, lymphoma linked to TNF inhibitors, leukemia, breast, colorectal, and prostate cancers.

Previous studies have shown that there is a significant association between RA and subsequent development of lymphoproliferative malignancy [4]. These malignancies include many different disease entities with distinct cells of origin, pathologies, risk factor profiles, and prognosis [5]. Lymphomas have been classified into Hodgkin's lymphoma (HL) and non-Hodgkin's lymphoma (NHL) including multiple subtypes such as follicular lymphoma (FL), leukemia (CLL), and lymphoplasmacytic lymphoma (LPL)/Waldenstrom's macroglobulinemia (WM). Although the causes of these lymphoproliferative malignancies are mostly unknown, data support a role for genetic and immune-related factors in their pathogenesis [6]. 

Earlier evidence has shown that there is an increased risk of serious infections and a dose-dependent increased risk of malignancy in RA patients treated with anti-TNF antibody therapy [7]. Although the malignancies reported in these trials are rare, comparing patients treated with anti-TNF therapies versus patients receiving methotrexate or no disease-modifying antirheumatic drugs suggested an increased risk of hematological malignancies in anti-TNF treated patients [8]. In addition, an increased risk of nonmelanoma skin cancers has been reported in patients with RA treated with anti-TNF agents in combination with methotrexate [9]. Furthermore, a dose-dependent increase in the risk of malignancies should be taken in account when considering anti-TNF antibody treatment in patients with RA. 

Estrogens and their metabolites play an important role in autoimmune rheumatic diseases [10] and cancer [11, 12]. Estrogen has not only anti-inflammatory but also proinflammatory roles depending upon different influencing factors [13]. Even the downstream metabolites of estrogen were shown to have important effects relevant to inflammation. Estrogen metabolites are also associated with different types of cancer, and these metabolites could provide a convenient marker either of increased risk of cancer or of early cancer, if altered levels precede the onset of clinical disease.

Currently, the exact causes of cancer morbidities in RA are mostly unknown; however, data support a role of estrogen metabolites in cancer morbidities in RA patients. In this paper, we will review the findings from studies on this topic and discuss the possible role of estrogen metabolites (catecholestrogen and other active metabolites) in different types of malignancies associated with RA. Furthermore, we discuss that the same mechanism is operated both in RA and cancer, as the data suggested the production of estrogen metabolites induced reactive oxygen species (ROS), which could modify DNA and produce different immune responses.

## 2. Estrogen Metabolites

Estrogen is a hormone group that comprises estrone, estradiol, and estriol. It has been hypothesized that the initiation of cancer may result from induction of DNA damage by estrogen metabolites, although progression may be facilitated by estrogen receptor-mediated upregulation of mitogenic genes. There are various reports that confirm that estrogen metabolites are tumorigenic in animal model [14, 15]. Estradiol and estrone undergo extensive oxidative metabolism via action of several cytochromes P450 (mainly CYP1A1 and CYP1B1). These are major enzymes catalyzing *β*-nicotinamide adenine dinucleotide phosphate (NADPH) dependent oxidative metabolism of estrogen to multiple hydroxylated metabolites. Each cytochrome P450 favours the hydroxylation of specific carbons, although, cytochrome enzymes can hydroxylate virtually all carbons with the exception of the inaccessible angular carbon. Liver microsomes from adult rats resulted in the formation of up to 20 detectable estrogen metabolites when incubated with [4-^14^C] estradiol and NADPH [16]. The generation of hydroxyl and keto functions at specific sites of the steroid nucleus markedly affects the biological properties of the respective estrogen metabolites whether the reaction yields estrogenic, nonestrogenic, or carcinogenic metabolites. Functionally, the most important reactions catalyzed by cytochrome P450 are at carbon 2, 4, and 16.

Catecholestrogens are biologically active metabolites of estrogen which are synthesized by enzyme 2- and 4-hydroxylase in the liver, brain, and other organs [17]. They are generated by actions of genes encoded by CYP1A1, CYP1A2 (which catalyze 2-hydroxylation of estrogens), and CYP1B1, which are having 4-hydroxylase activity. Specifically, CYP1A1 converts estradiol firstly to 2-hydroxyestradiol (2-OHE_2_) and then to the estradiol-2,3-semiquinone (E-2,3-SQ) and estradiol quinone (E-Q). CYP1B1 converts estradiol firstly to 2- and 4-hydroxyestradiol (4-OHE_2_) and then to corresponding semiquinone and quinones. 4-Hydroxyestrogens can oxidize to quinone intermediates that can react with purine bases of DNA, resulting in depurinating adduct that can generate highly mutagenic apurinic sites. Quinones derived from the 2-hydroxyestrogen produce stable DNA adducts and are presumed to be less genotoxic [18]. The catecholestrogens also serve as substrate for catechol-o-methyl transferase (COMT), which catalyze o-methylation by forming monomethyl ethers at the 2-, 3-, and 4-hydroxyl groups [19]. COMT generated two products from 2-hydroxyestrogens in contrast to the single product from 4-hydroxyestrogens [20]. However, small amount of catecholestrogen may be converted by peroxidase-catalyzed reaction to yield semiquinone or quinones that are capable of forming DNA adducts or of generating ROS that could oxidize DNA bases [19]. 4-OHE_2_ generates free radicals through reductive-oxidative cycling, thus causing DNA damage. In contrast to 4-hydroxyestrogens, 2-hydroxyestrogen is not carcinogenic and has potent inhibitory effect on the growth of tumor cells and on angiogenesis [21].

In human, cytochrome P450 3A7 has strong catalytic activity for estrone 16 *α*-hydroxylation [22]. Similar to the catecholestrogen, the 16 *α*-hydroxylated estrogens are hormonally active, chemically reactive, and potentially mutagenic. 16 *α*-hydroxyestrone (16 *α*-OHE_1_) possesses the unique property of binding covalently to the estrogen receptor and other nuclear proteins, such as histones. Levels of 16 *α*-OHE_1_ can rise in response to obesity, alcohol consumption, and toxic exposure. High levels of this potent metabolite are linked to increased risk and poorer prognosis in conditions associated with estrogen excess, including cancer and lupus.

As discussed already, the major metabolites of estradiol and estrone are those hydroxylated at either the C-2 or the C-16 positions. Hydroxylated metabolites at the C-4 position are also present, but in lesser amount. However, the C-4 and C-16 hydroxylated estrone or estradiol metabolites are different from C-2 because these metabolites have more estrogenic activity than that of their mother compound [23]. It has been suggested that women who metabolize a large proportion of their estrogen down the C-4 pathway, in contrast to the C-2 pathway, have elevated breast cancer rates, while the daughter estrogen metabolized down the C-16 route may be associated with a direct genotoxic effect and carcinogenicity [24]. Studies have found that if particular enzymes within cytochrome family, namely, P450 1A1 and 1A2, are activated, then more parent estrogens are metabolized into C-2 hydroxylated compounds [25]. However, if cytochrome P450 3A4 and 1B1 are activated, then more C-4 and C-16 are produced [26]. 

## 3. Estrogen Metabolites and Mutations in DNA 

Endogenous estrogen can become carcinogenic via formation of catecholestrogen quinones, which react with DNA to form specific depurinating estrogen-DNA adducts. Catalytic oxidation of the catecholestrogens (2-hydroxy and 4-hydroxyestrogens) gives rise to the corresponding estrogen-2,3-quinone (E-2,3-Q) and estrogen-3,4-quinone (E-3,4-Q), which react with DNA to form adducts [27]. These adducts can form stable mutations that remain in the DNA unless they are corrected by repair. Alternatively, the modified bases can be released from DNA by destabilization of the glycosidic bond and result in formation of depurinating or depyrimidinated sites [27]. 

Few studies have reported the presence of catecholestrogen adduct in human breast tissues [28, 29]. It has been found that every DNA sample from these tissues consists of deoxyguanosine adducts of 4-OHE_2_ and 4-OHE_1_. The formation of catecholestrogen quinone-derived DNA adducts has also been reported in these two breast samples [29], and the adducts identified were 4-hydroxyestradiol-1-N3-adenosine, 4-hydroxyestrone-1-N3-adenosine, and 4-hydroxyestradiol-1-N7-guanine. Treatment of E-3,4-Q with dG also results in the formation of 4-hydroxyestradiol-1-N7-guanosine [30]. The reaction of E-3,4-Q with dG at the N-7 position destabilizes the glycosidic bond and results in loss of the deoxyribose moiety. Once the adduct is formed by reaction of E-3,4-Q with DNA, it is released from the DNA by spontaneous depurination. In addition, injection of 4-OHE_2_ or E-3,4-Q in mammary glands of female ACI rats resulted in formation of the depurinating adducts 4-hydroxyestradiol-1-N3-adenosine and 4-hydroxyestradiol-1-N7-guanosine [31]. Reaction of E-3,4-Q with dA produced no adducts; however, its reactions with Ade resulted in the formation of 4-hydroxyestradiol-1-N3 Ade [32]. When E-3,4-Q react with dG or dA, the adducts formed were identified as 2-hydroxyestradiol-6-N^2^ dG and 2-hydroxyestradiol-6-N^6^ dA. DNA adducts derived from 2-hydroxyestrogen-quinone have been shown to be mutagenic and primarily produced G-T and A-T mutations in Simian kidney cells [33]. In contrast to 2-hydroxyestrogen, E-3,4-Q reacts rapidly to form 4-hydroxyestradiol-1-N3-adenosine adducts that are depurinating adducts. Numerous A→G mutations in H-ras DNA were observed in SENCAR mouse skin treated with E-3,4-Q [34].

Estrogens induce lipid peroxidation during their metabolic activation [35]. Lipid hydroperoxides formed during estradiol metabolism may serve as cofactors in further estradiol metabolism to hydroxylated products and in the oxidation of catecholestrogen to quinone intermediates. In addition, lipid hydroperoxide-derived aldehydes (malondialdehyde and 4-hydroxynonenal) interact with bases in cellular DNA, thus increasing the burden of DNA modification [36]. Oxidative metabolism of estrogen metabolites produced three types of DNA damage: DNA base adducts produced by quinone, as describe above, lipid hydroperoxide-derived aldehyde DNA adducts, and plethora of oxidized DNA bases.

Earlier studies from our lab showed that estrogen metabolites (especially catecholestrogen) cause single and double strand breaks, change in the ellipticity of the double helical DNA, and various other types of DNA modifications [11, 37–40]. It is interesting to note that modification induced by these catecholestrogen metabolites was the greatest for thymine followed by guanine, adenine, and cytosine. In addition, catecholestrogen metabolites cause more extensive damage to DNA in presence of copper in comparison to nitric oxide. These results demonstrate that catecholestrogen leads to the production of potent ROS, which is capable of causing DNA damage, thus playing important role in carcinogenesis [11, 12] and various autoimmune diseases [37–40].

Mutations are caused by numeric changes or structural alterations in the genome. Recent study has shown that some estrogen metabolites are capable of producing genome instability [41]. DNA modification by these reactive estrogen metabolites might explain some of the structural and numeric chromosomal changes observed in response to estrogen exposure [42]. In conclusion, estrogen metabolites can produce multiple types of genetic insults contributing to the induction of genomic instability. 

## 4. Estrogen Metabolites and Immune System

Sex hormones are implicated in the immune response, with estrogen as enhancer of humoral immunity and androgens as natural suppressor of the immune response [43]. Sex biases in autoimmunity and infection, together with immune cell expression of estrogen and androgen receptors, suggest that sex steroid hormone directly modulates immune cells [44]. Dual proinflammatory and anti-inflammatory effects of estrogen in human and experimental inflammation depend on various factors including intracellular conversion of estrogen into active metabolites with quite different pro-inflammatory and anti-inflammatory functions [13]. A pro-inflammatory microenvironment leads to conversion of androgen to estradiol and downstream estrogens [45], that are further converted to 16-hydroxylated active estrogens but not to 2-hydroxylated endogenous antiestrogens [46]. A shift from 2-hydroxyestrogens to 4-hydroxyestrogens might be an additional pro-inflammatory signal because these estrogen metabolites can be converted to 3,4-quinones, which are capable of damaging DNA leading to depurination and mutation in vivo [31]. Similarly, a shift to 16 *α*-hydroxylated forms of estrogen can be an important pro-inflammatory and proliferative signal [10, 13]. In contrast to the prooxidant activities of 4-OHE_2_, 2-OHE_2_ is a potent antioxidant that prevents membrane phospholipids and cells against ROS [47]. Thus 4-OHE_2_ in comparison to 2-OHE_2_ induces carcinogenic and pro-inflammatory effects [48]. Presently, we do not know whether pro-inflammatory mediator induces a shift from 2-hydroxyestrogen to 4- and 16-hydroxylated estrogens. 

Estradiol increased IgG and IgM production by peripheral blood mononuclear cells (PBMC) in patients who had systemic lupus erythematosus (SLE). This led to elevated levels of polyclonal IgG, including IgG anti-dsDNA, by enhancing B-cell activity via IL-10 [49]. Estrogen can also modulates pro-inflammatory cytokines released from activated monocytes or macrophages, in particular through the modulation of CD 16 expression [50]. It was recently shown that disease activity in patients who had SLE correlated negatively with urinary concentration of 2-hydroxylated estrogens [10]. Hence estrogens are confirmed as one of the risk factors for autoimmunity. 

The possible roles of downstream metabolites of estrone and estradiol were shown to have important effects relevant to inflammation. The growth inhibiting effects of 2-OHE_2_ and 2-methoxyestradiol come into limelight, several of these effects are independent of ERs, and relatively high concentrations of hormone were used [46, 48]. 2-Methoxyestradiol inhibited endothelial cell proliferation and migration as well as angiogenesis in vitro [51]. Because neoangiogenesis is an important mechanism in inflammation, *α*-methoxyestradiol can exert anti-inflammatory activities by inhibiting vessel formation. These lines of evidence showed that 2-methoxyestradiol is a proapoptotic and cytostatic endogenous compound, which can inhibit neoangiogenesis and can attenuate inflammation. In contrast, 4-hydroxylated estrogens might exert pro-inflammatory roles by inducing ROS and DNA damage. 

One of the important properties of estradiol is its ability to differentiate T and B cells, increase immunoglobulin production, and aggravate immune complex mediated diseases [52]. Immunoglobulin production was also seen in those patients who are diagnosed with breast cancer [53]. These studies are in agreement with our previous studies that explain the presence of antibodies against catecholestrogen-modified DNA (CE-DNA) in the sera of cancer [11, 12], SLE [37–39], and RA patients [40]. Studies from our laboratory demonstrate that catecholestrogens are likely to contribute to the generation of antibodies which may directly or indirectly affect the immune system.

Estrogen and some of its metabolites (estradiol, estrone, 16 *α*-OHE_1_, and estriol) may induce myeloperoxidase release from the resting cells and stimulate generation of oxidants [54]. 2-hydroxylated estrogen acts as powerful inhibitors of PMNs (polymorphonuclear leukocytes, neutrophils, and granulocytes) activity, which show protective properties of the 2-hydroxylated catecholestrogen. In addition, estrogen causes macrophage proliferation and activation, and in turn macrophages produce estrogens, which may act on other phagocytic cells. Hence, macrophages activation may lead to ROS production, which may cause DNA base hydroxylation, oxidation, nitration, and deamination. In conclusion, estrogens and their metabolites affect inflammatory responses, and in turn, their activities are controlled by different inflammatory products.

## 5. Role of Estrogen Metabolites in Cancer Morbidity in RA

Patients with RA have double the risk of death from the disease compared to the general population. Conclusions about whether RA patients are more likely to die from cancer than the general population have been less clear. Many studies have confirmed that although rates of over all cancer are not increased substantially from the general population, certain types of cancers may be seen with higher frequency in patients with RA [9, 55]. Most commonly, an increased risk for development of lymphoproliferative disorders, particularly non-Hodgkin's lymphoma, has been shown in patients with RA [56]. It is unclear whether the increased risk is due to aberrancies in the immune system from higher inflammatory activity in RA, from certain immunosuppressive agents used to treat RA, or from a combination of the both [57]. It has been suggested that an increase risk for the development of nonmelanoma skin cancers (NMSC), such as basal cell carcinoma (BCC) and squamous cell carcinoma (SCC), was observed in patients with RA when compared to its rate in the general population [58]. The association between RA and malignancies has received increased attention in recent years. Reports suggested that tumor necrosis factors (TNFs) blockers might elevate the risk of malignancy in RA patients [58]. There is a relationship between the level of inflammation in RA patients and their risk of developing lymphoma. This risk might be magnified by knowing the various roles played by different types of estrogen metabolites in cancer morbidities in RA. Overall, the clinical relevance of these data regarding RA and malignancy is unclear. 

Estrogen metabolites are known to play an important roles in various autoimmune diseases (especially RA) [10, 37–40, 59] and cancer [11, 12]. In patients with RA and SLE, urinary concentration of the downstream mitogenic 16 *α*-OHE_1_ and 2-OHE_1_ or naturally occurring antiestrogen was investigated. These studies have shown that urinary concentrations of the 2-hydroxylated estrogens were 10 times lower in patients with RA and SLE than those in healthy control, whereas the ratio of 16 *α*-OHE_1_/2-hydroxyestrogen was 20 times higher in RA and SLE patients than that in control [10]. These results showed that the magnitude of conversion to the mitogenic 16 *α*-OHE_1_ is extremely upregulated in RA and SLE. Testing for urinary levels of 2-OHE_1_ and 16 *α*-OHE_1_ provides valuable insight regarding the assessment for cancer risk [60]. The ratio of 2-OHE_1_ to 16 *α*-OHE_1_ (or estrogen metabolic index (EMI)) should be greater than 2 and values in the upper normal range are advisable. Women with breast and endometrial cancers have marked elevation of 16 *α*-OHE_1_, which is a significant risk factor for estrogen-dependent tumor [61]. Tumors in other estrogen-sensitive tissues are also linked to 16 *α*-OHE_1_. It has been found that the ratio of 2-OHE_1_ to 16 *α*-OHE_1_ is a risk factor not only for breast cancer but also for other conditions of inappropriate estrogen activity. 16 *α*-OHE_1_ is a mitogenic and proliferative endogenous hormone that covalently binds to estrogen receptor leading to nuclear translocation [62]. This metabolite is converted from upstream estrone and estradiol, and because of this covalent linkage to the receptor, it shows persistent biological responses consisting of mitogenic tumor stimulation [63]. 2-OHE_1_ is essentially devoid of peripheral biological activity, has been found to exert a modest antiestrogenic effect [60], and considered as “good estrogen” [64]. In addition, 16 *α*-OHE_1_ has been found to be elevated in those at risk for breast cancer as well as other conditions associated with hyperimmune activity such as RA and SLE. In these conditions, 16 *α*-OHE_1_ was 10 times higher in the control population [10]. Therefore, estrogen metabolism should be taken into consideration when treating patients with autoimmune conditions.

Elevated serum concentration of 16 *α*-OHE_1_ has been described in patients with SLE [65], suggesting that abnormal patterns of estradiol metabolism may lead to increased estrogenic activity. A similar phenomenon was recently described in the synovial fluids (SFs) of RA patients, where 16 *α*-OHE_1_ and 4-OHE_2_ were found to be significantly higher compared with control fluid [45]. Interestingly, women who developed knee osteoarthritis, which is also an inflammatory disease, were more likely to have a ratio of 16 *α*-OHE_1_ to 2-OHE_1_ in highest tertile and 2-hydroxyestrogen in the lowest tertile [66]. The loss of 2-hydroxyestrogen is obviously a pro-inflammatory signal, which might be unfavorable in breast cancer development. Catecholestrogen, 2-OHE_2_, and 4-OHE_2_ have been demonstrated to induce uterine adenocarcinoma in the CD-1 mouse model [14]. 2-Hydroxylated metabolites of estrogen have been shown to have antiangiogenic effects and inhibit tumor cell proliferation, whereas 4-hydroxylated metabolites are known to be implicated in carcinogenesis. In addition, estradiol and 4-OHE_2_ are mutagenic even at lowest dose, whereas 2-OHE_2_ induced mutation at very high concentration. In animal model, 4-OHE_2_ had a carcinogenic effect equal to that of estradiol, whereas 2-OHE_2_ did not induce kidney tumors in hamsters [67] and had much less ability to induce uterine adenocarcinoma in CD-1 mice than did 4-OHE_2_ [14]. Furthermore, the formation of depurinating adducts, by the reaction of E-3,4-Q enzymes-catalyzed oxidation of 4-OHE_2_ with DNA, is much higher than that from reaction of E-2,3-Q enzymes-catalyzed oxidation of 2-OHE_2_ with DNA [68]. Interestingly, the 4-hydroxylated estrogens were 3.5 times more abundant than the 2-hydroxylated estrogen in women with breast cancer and were 4 times higher in women without breast cancer [69]. The rate of 4-OHE_2_ formation exceeded those of 2-OHE_2_ formation by almost 4 fold in mammary fibroadenomas and adenocarcinoma, although the rate did not markedly differ in normal human mammary tissues [15]. 

Besides various studies investigating urinary concentration of the downstream mitogenic estrogen metabolites (16 *α*-OHE_1_, 2-OHE_2_, and 4-OHE_2_) in cancer [60] and autoimmune diseases [10], data from our laboratory demonstrate that estrogen metabolites especially catecholestrogens (2- and 4-hydroxylated estrogens) play important role in cancer [11, 12] as well as in various autoimmune diseases including RA [37–40]. It has been found that CE-DNA was highly recognized not only by circulating cancer autoantibodies [11, 12] but also by RA [40] and SLE autoantibodies [37–39], indicating the possible participation of CE-DNA in the pathogenesis of cancer and RA. It is interesting to note that single antigen (CE-DNA) is known to play dual role in the etiopathogenesis of RA and cancer, and that might be the reason that cancer morbidities are common in RA. Since native DNA is a known weak immunogen, it appears that DNA damage by catecholestrogen renders it immunogenic leading to the induction of RA and cancer autoantibodies. The recognition of CE-DNA by autoantibodies in cancer and RA was further evaluated by quantitative precipitin titration, and it was found that high values of affinity constant clearly indicate better recognition of CE-DNA by these circulating cancer and RA autoantibodies [11, 12, 40], and hence prove the hypothesis that malignancies are associated with RA. The intracrine synthesis of active estrogen metabolites at the level of cells was involved in the immune response (e.g. synovial macrophages and fibroblasts) [70]. In addition, recent findings indicate that RA synovial cells mainly produce the proproliferative 16alphaOH-estrone, which, in addition to 16alphaOH-17beta-estradiol, is one of the only 2 estrogens studied that does not inhibit TNF secretion. A preponderance of 16alpha-hydroxylated estrogens is an unfavorable sign in synovial inflammation. Women at high risk of breast cancer, those with benign breast disease and those who are diagnosed with breast cancer, have significantly elevated anti-HMdU (5-hydroxymethyl-2′-deoxyuridine) autoantibodies [53]. The presence of high anti-HMdU autoantibodies levels explains the prooxidant conditions that have lead to oxidation of bases in cellular DNA and have evoked an autoimmune response just like RA in our previous study [40]. These reports suggest that the oxidative DNA base damage and the biologic responses it evokes start occurring not only in carcinogenesis but also in RA. These results are further strengthened by data obtained from our study and by other investigators that showed that oxidative DNA base damage is evident in DNA of individuals who are at risk for hormone-dependent cancers [53] and RA [40]. These studies show that estrogen metabolites might play important role in cancer morbidities in RA.

## 6. Conclusion

**Figure 1:**
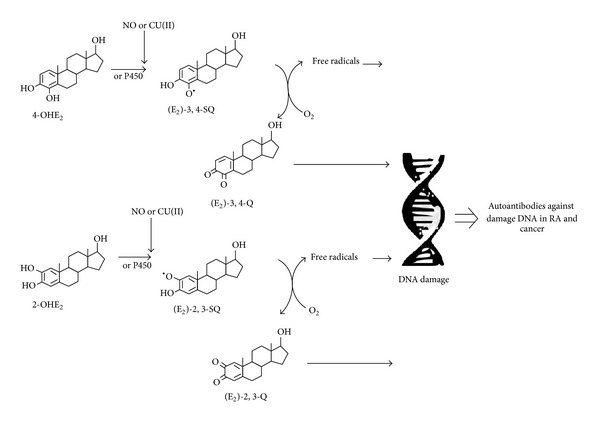
The proposed mechanism for cancer morbidity in RA. 4-OHE_2_ (4-hydroxyestradiol), 2-OHE_2_ (2-hydroxyestradiol), (E_2_)-3,4-SQ (estradiol-3,4-semiquinone), (E_2_)-2,3-SQ (estradiol-2,3-semiquinone), (E_2_)-3,4-Q (estradiol-3,4-quinone), and (E_2_)-3,4-Q (estradiol-2,3-quinone).

The association between RA and malignancies has been well established, but the exact mechanism for carcinogenesis in RA is lacking. Estrogen metabolites seem to play an important role in RA and cancer, but the mechanisms that connect these two conditions are missing. It is obvious that we must consider not only the parent estrogen when we evaluate disease risk (RA and cancer) but also the estrogen metabolites. While we do not have the full picture of all the responsible factors involved in cancer morbidity in RA, data indicate that oxidative reactions, catalyzed by isoform of the cytochrome P450, can result in the formation of catecholestrogens from the parent compound, and quinone/semiquinone redox cycle of the catecholestrogen metabolites is capable of forming either stable or depurinating DNA adducts. Oxidation of these estrogen metabolites also leads to high amount of ROS that can cause extensive DNA damage. This would probably alter its immunogenicity leading to the induction and elevated levels of RA and cancer autoantibodies (Figure 1). Estrogen is known to play both pro- and anti-inflammatory roles that were found to be related to the intracellular conversion into active metabolites with different pro- and anti-inflammatory functions. Particular lines of evidence related to estrogen administrate seem to support those effects. Since 17beta-estradiol administered during hormone replacement therapy (i.e. early menopause, endometriosis, etc...) in RA patients will rapidly increase estrone sulfate after conversion in adipose tissue by aromatases, hormone replacement therapy can have proinflammatory and cell proliferative effects by providing estrone sulfate to the inflamed synovial tissue. In addition, it appears that the use of combined oral contraceptives is associated with an increased risk of at least systemic lupus erythematosus (disease flare up, worsening, etc...) [71]. So estrogen modulates immune response by affecting various inflammatory responses, and in turn, their activities are affected by these inflammatory products. Only by understanding the complex interaction of various estrogen metabolites and mechanism behind these two pathological conditions, we will be able to manage cancer morbidities in RA.
